# A case report of granulomatous polyangiitis complicated by tuberculous lymphadenitis

**DOI:** 10.1097/MD.0000000000012430

**Published:** 2018-10-26

**Authors:** Yuki Iijima, Yoichi Kobayashi, Yoshinori Uchida, Toshiharu Tsutsui, Yumiko Kakizaki, Tsukasa Naganuma, Katsuhiko Tsukamoto, Toshio Oyama, Yoshihiro Miyashita

**Affiliations:** a Lung Cancer and Respiratory Disease Center, Yamanashi Central Hospital; b Nephrology, Yamanashi Central Hospital; c Dematorogy, Yamanashi Central Hospital; d Pathology, Yamanashi Central Hospital, Kofu City, Yamanashi, Japan.

**Keywords:** eosinophilia, granulomatous polyangiitis, PR3-ANCA, tuberculosis

## Abstract

**Rational:**

Granulomatous polyangiitis (GPA) is a type of vasculitis involving medium and small arteries, typically affecting the upper and lower respiratory tract with coexisting glomerulonephritis. GPA is also characterized by necrotizing granulomatous inflammation and the presence of antineutrophil cytoplasm antibodies (ANCA). So far, various infections have lead to elevation of titers of serum ANCA, making it difficult to diagnose.

**Patient Concerns:**

We report a 50-year-old woman who was diagnosed as tuberculous lymphadenitis. During the treatment by anti-tuberculosis (TB) drugs, rapidly progressive renal failure and pleurisy had appeared with elevated titer of PR3-ANCA. Renal biopsy revealed crescentic glomerulonephritis.

**Diagnosis:**

Renal biopsy revealed crescentic glomerulonephritis and diagnosis of GPA was made.

**Interventions:**

Steroid therapy had been started with continuation of anti-TB drugs.

**Outcomes:**

Renal dysfunction had gradually recovered and pleurisy had disappeared with decreasing titer of PR3-ANCA.

**Lessons:**

This is the first report of GPA complicated by TB infection. When we encounter a case with rapidly progressive renal failure during the TB infection, complication of GPA should be suspected as 1 of the different diagnosis.

## 1 Introduction

Granulomatous polyangiitis (GPA) is a type of vasculitis involving medium and small arteries, typically affecting the upper and lower respiratory tract with coexisting glomerulonephritis. GPA is also characterized by necrotizing granulomatous inflammation and the presence of antineutrophil cytoplasm antibodies (ANCA).^[1]^ However, some infectious diseases, such as bacterial endocarditis may sometimes show high titers of ANCA, mimicking vasculitis.^[2,3]^ Moreover, such infectious diseases are occasionally associated with vasculitis as a genuine complication.^[4]^ Even in cases of GPA, an association with infectious disease has been reported; for example, chronic carriage of *Staphylococcus aureus* might be a risk factor for relapsing disease.^[5]^ However, how these conditions are associated with each other remains unknown. Here, we describe a case of GPA complicated by tuberculous lymphadenitis, in which several etiologies may be considered to explain this complication.

## 2 Case report

**Figure 1:**
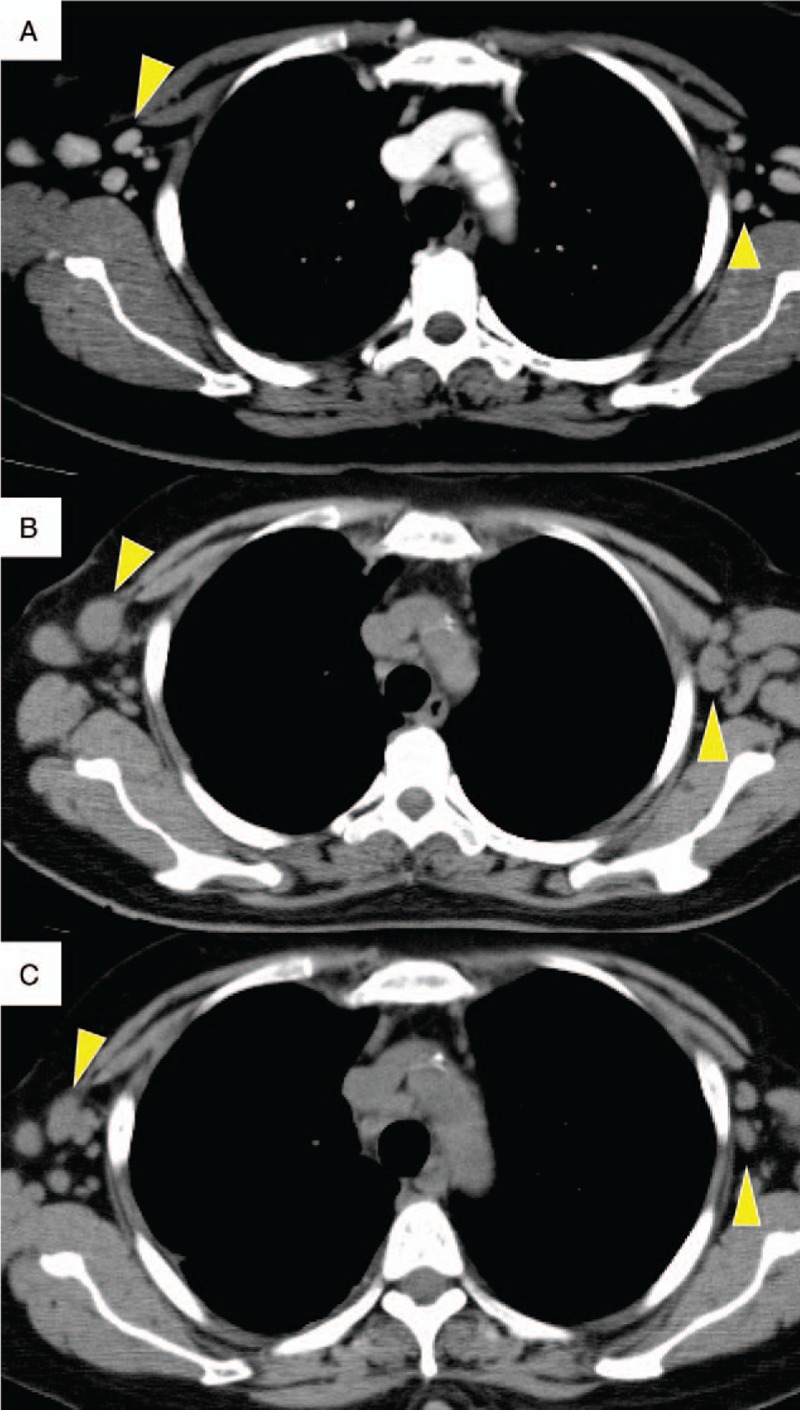
Size change in lymphadenopathy. At the first visit to our hospital. Lymphadenopathy was seen at the axillary and cervical lymph nodes. Just before continuous use of anti-TB drugs. Enlargement in the lymphadenopathy was observed. Size of the axillary lymphadenopathy showed remission after continuous use of anti-TB drugs. TB =  tuberculosis.

A 50-year-old Filipino woman presented with nodular erythema on the arms, legs, and face. She had no history of allergy or medications and had no past medical history such as bronchial asthma. One year after initial presentation, a dermatologist performed a skin biopsy, wherein histopathological findings showed eosinophilic infiltration. Blood examination showed eosinophilia (3450/μL; normal, <500 /mm^3^) and abnormally elevated levels of nonspecific IgE (113,000 IU/mL; normal, <170 IU/mL) and Th-2 chemokine (TARC) (27,480 pg/mL; normal, <450 pg/mL). As T-SPOT test was positive, *Mycobacterium tuberculosis* infection was suspected. Therefore, she was referred to our hospital for further investigation. Computed tomography (CT) findings did not show infectious lesion in the lung fields but showed swollen lymph nodes on both sides of the axillae and the neck (Fig. 1A). *M tuberculosis* was cultured from the axillary lymph node biopsy specimen, and the patient was accordingly diagnosed as having tuberculous lymphadenitis.

Anti-tuberculosis (TB) drugs were started as a combination protocol of isoniazid, rifampicin, ethambutol, and pyrazinamide. However, the patient experienced nausea and edema, and she had to stop the treatment only 5 days after initiation. Two months had passed after stopping the therapy because the patient dropped out from attending our hospital. The axillary lymphadenopathy worsened, and the lymph nodes further increased in size (Fig. 1B). As rifampicin was suspected to be the causative agent of the previous symptoms, anti-TB therapy was restarted with isoniazid, ethambutol, and pyrazinamide. However, the patient developed renal dysfunction. The drugs were stopped again 83 days after the second initiation. In spite of cessation of drug administration, the renal dysfunction worsened, and she was admitted to the hospital.

**Figure 2:**
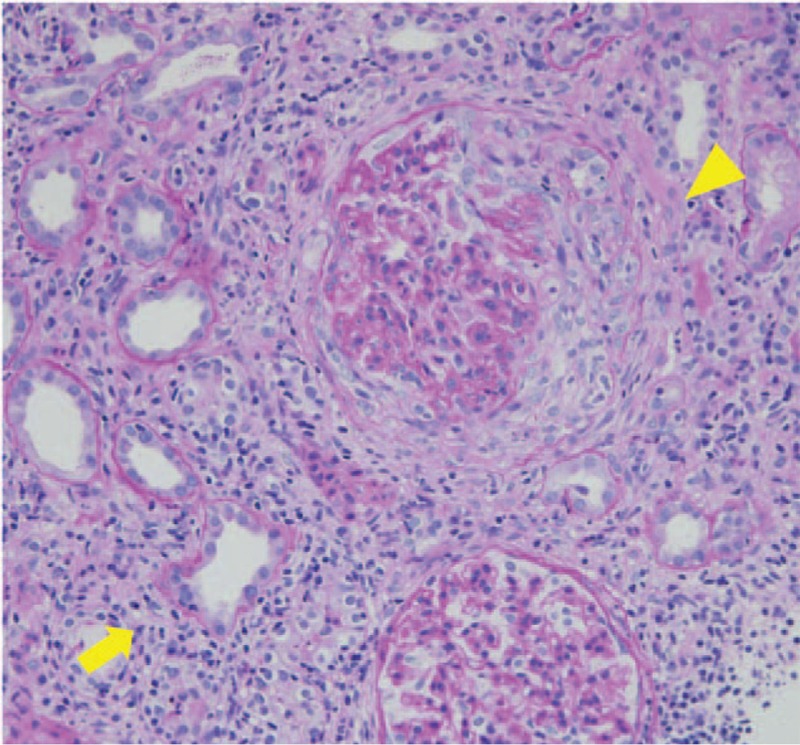
Pathological findings of kidney biopsy. Kidney biopsy revealed crescentic glomerulonephritis (arrow head) and peritubulitis (arrow), concordant with GPA. GPA = granulomatous polyangiitis.

**Figure 3:**
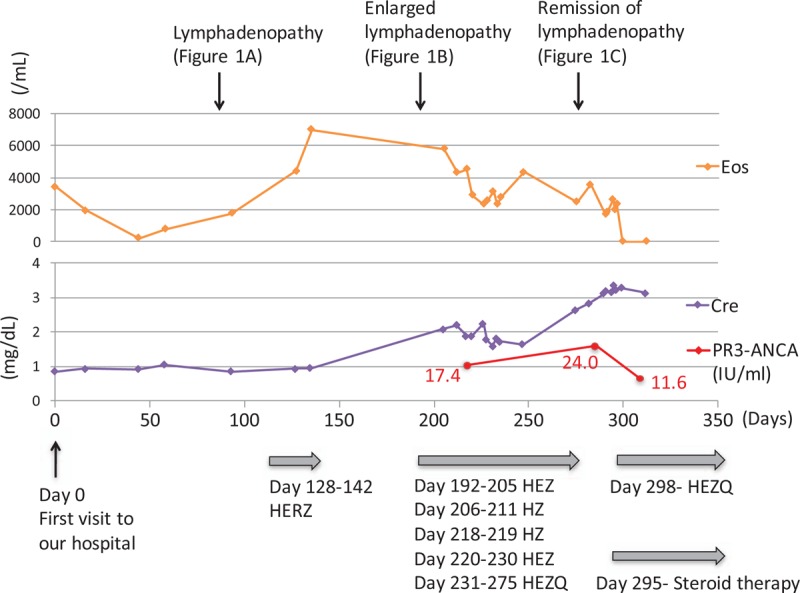
Clinical course after visiting our hospital. After initiating anti-TB treatment, eosinophils counts decreased with remission of lymphadenopathy. However, renal dysfunction worsened with elevated PR3-ANCA and newly emerged pleurisy. ANCA = antineutrophil cytoplasm antibodies, Cre = creatinine, E = ethambutol, Eos = eosinophils, H = Isoniazid, Q = levofloxacin, Z = pyrazinamide.

On admission, vital signs were almost normal: blood pressure, 160/100 mmHg; pulse rate, 102 beats/min; body temperature, 36.8°C; and SpO_2_, 98% (room air). Physical examination showed no abnormal signs other than the presence of nodular papules on the face. Laboratory findings revealed eosinophilia, with a count of 1690/mm^3^ (normal, <500/mm^3^), which was still high but lower than before starting anti-TB treatment. The serum creatinine level was 3.11 mg/dL (normal, <1.0 mg/dL), and C-reactive protein was 3.43 mg/dL (normal, <0.3 mg/dL), with an erythrocyte sedimentation rate (ESR) of 123 mm (normal range, 2–10 mm). The serum PR3-ANCA level was elevated to 24.0 U/mL (normal, <3.5 U/mL). Urinalysis showed hematuria (3+) and proteinuria (2+). Chest CT showed regression of axillary and cervical lymphadenopathy, reflecting the efficacy of previous anti-TB therapy (Fig. 1C). However, new pleural effusion was evident on the right side and no mycobacteria were cultured from the pleural fluid sample. Renal biopsy was performed, and histopathological examination revealed crescentic glomerulonephritis with peritubulitis (Fig. 2). Otolaryngological medical examination also revealed right chronic otitis. Based on these findings, we diagnosed GPA; pleurisy, otitis, and renal failure were considered to be organ disorders subsequent to vasculitis. Accordingly, 500 mg of methylprednisolone was administered once daily for 3 days followed by 40 mg of prednisone for 2 weeks. Eosinophil counts decreased to undetectable levels along with resolution of the rash and pleurisy, and the PR3-ANCA level decreased to 11.6 U/mL (normal, <3.5 U/mL). The serum creatinine level also gradually decreased (Fig. 3). In addition, anti-TB treatment was restarted with isoniazid, ethambutol, pyrazinamide, and levofloxacin. The treatment had been continued for 18 months, and neither any adverse event nor relapse of TB lymphadenopathy had occurred after that.

## 3 Discussion

Here, we have described a case of GPA complicated by tuberculous lymphadenitis. So far, various infections have been known to lead to secondary elevation of serum ANCA.^[2–4]^ Even among patients with TB, it is well known that serum ANCA titers are higher than those in normal people.^[6–10]^ These factors make it difficult to diagnose the complication of vasculitis. However, in this case, remission of lymphadenopathy was noted after initiating the anti-TB therapy, while the serum PR3-ANCA titer remained high, with worsening of organ dysfunction including renal failure and pleurisy of unknown etiology. This clinical course could not be explained only by a systemic TB-induced condition alone, and it indicated the presence of vasculitis as a complication. Previously, certain types of vasculitis, such as cutaneous leukocytoclastic vasculitis and Henoch-Schönlein purpura, have been reported to be accompanied by TB.^[11,12]^ However, with regard to the relationship between TB and GPA, only 2 case reports are available in which GPA mimicked TB or vice versa.^[13,14]^ Thus, this is the first report to show GPA complicated with TB.

The etiology underlying the concurrence of TB and GPA remains unclear and this is a limitation of this case. The most plausible etiology was “anti-TB drug-induced vasculitis”. Previously, various agents have been reported as causative drugs for vasculitis, such as propylthiouracil (PTU) and minomycin.^[15,16]^ It is speculated that these drugs might interrupt the resolution of the neutrophil extracellular trap, acting as the mechanism underlying the accompanying vasculitis. Among anti-TB drugs, Carmela et al reported a case in which isoniazid used for latent TB infection induced ANCA-related vasculitis.^[17]^ Kim et al also reported a case of cutaneous leukocytoclastic vasculitis due to rifampicin and pyrazinamide.^[18]^ The other speculation explaining the etiology is that secondary elevation of ANCA induced by a chronic inflammation by TB infection lead to development of GPA.

The presence of eosinophilia was also interesting findings in this case. Generally, mycobacterial infections induce a Th-1 type immune response.^[19,20]^ However, in this case, eosinophilia existed with abnormally high levels of IgE and TARC, which improved after the initiation of anti-TB therapy. The patient had no previous history of bronchial asthma or atopy, and no history of taking causative medications, including supplements. There were no findings indicating allergic bronchopulmonary aspergillosis or malignancy. One probable speculation is that allergic reaction to TB body might have existed and resulted in Th-2 type immune response.

In conclusion, this is the first case report of GPA complicated by tuberculous lymphadenitis. When we encounter a case of renal failure appearing with TB infection, GPA should be considered as 1 of the differential diagnoses.

## Author contributions

**Writing – original draft:** Yuki Iijima.

**Writing – review & editing:** Yoichi Kobayashi, Yoshinori Uchida, Toshiharu Tsutsui, Yumiko Kakizaki, Tsukasa Naganuma, Katsuhiko Tsukamoto, Toshio Oyama, Yoshihiro Miyashita.

## References

[1] Fahey J Leonard E Churg H Wegener's granulomatosis. Am J Med 1954;17:168–79. 13180525 10.1016/0002-9343(54)90255-7

[2] Mahr A Batteux F Tubiana S IMAGE study group. Brief report: prevalence of antineutrophil cytoplasmic antibodies in infective endocarditis. Arthritis Rheumatol 2014;66:1672–7. 24497495 10.1002/art.38389

[3] Ying CM Yao DT Ding HH Infective endocarditis with antineutrophil cytoplasmic antibody: report of 13 cases and literature review. PLoS One 2014;9:e89777. 24587028 10.1371/journal.pone.0089777 PMC3934949

[4] Guillevin L Mahr A Callard P Hepatitis B virus-associated polyarteritis nodosa: clinical characteristics, outcome, and impact of treatment in 115 patients. Medicine (Baltimore) 2005;84:313–22. 16148731 10.1097/01.md.0000180792.80212.5e

[5] Zycinska K Wardyn KA Zielonka TM Chronic crusting, nasal carriage of *Staphylococcus aureus* and relapse rate in pulmonary Wegener's granulomatosis. J Physiol Pharmacol 2008;59:825–31. 19218710

[6] Flores-Suárez LF Cabiedes J Villa AR Prevalence of antineutrophil cytoplasmic autoantibodies in patients with tuberculosis. Rheumatology (Oxford) 2003;42:223–9. 12595614 10.1093/rheumatology/keg066

[7] Sherkat R Mostafavizadeh K Zeydabadi L Antineutrophil cytoplasmic antibodies in patients with pulmonary tuberculosis. Iran J Immunol 2011;8:52–7. 21427496 10.22034/iji.2011.16888

[8] De Clerck LS Van Offel JF Smolders WA Pitfalls with anti-neutrophil cytoplasmic antibodies (ANCA). Clin Rheumatol 1989;8:512–6. 2515023 10.1007/BF02032106

[9] Pradhan VD Badakere SS Ghosh K Spectrum of anti-neutrophil cytoplasmic antibodies in patients with pulmonary tuberculosis overlaps with that of Wegener's granulomatosis. Indian J Med Sci 2004;58:283–8. 15286419

[10] Esquivel-Valerio JA Flores-Suárez LF Rodríguez-Amado J Antineutrophil cytoplasm autoantibodies in patients with tuberculosis are directed against bactericidal/permeability increasing protein and are detected after treatment initiation. Clin Exp Rheumatol 2010;28:35–9. 20412700

[11] Maurício C Robson LD Denise S Cutaneous leukocytoclastic vasculitis accompanied by pulmonary tuberculosis. J Bras Pneumol 2008;34:745–8. 18982211 10.1590/s1806-37132008000900014

[12] Kitamura H Shimizu K Takeda H A case of Henoch-Schönlein purpura nephritis in pulmonary tuberculosis. Am J Med Sci 2007;333:117–21. 17301592 10.1097/00000441-200702000-00010

[13] Flores-Suárez LF Saldarriaga Rivera LM Rivera Rosales RM Cavitary tuberculosis and tracheal stenosis simulating granulomatosis with polyangiitis. Int J Tuberc Lung Dis 2015;19:369–70. 25692937 10.5588/ijtld.14.0633

[14] Mahmood FS Schwatz E Kurrup S A diagnostic dilemma: differentiating between granulomatosis with polyangiitis and tuberculosis. Clin Med (Lond) 2013;13:411–3. 23908518 10.7861/clinmedicine.13-4-411 PMC4954315

[15] Yamauchi K Sata M Machiya J Antineutrophil cytoplasmic antibody positive alveolar haemorrhage during propylthiouracil therapy for hyperthyroidism. Respirology 2003;8:532–5. 14708556 10.1046/j.1440-1843.2003.00499.x

[16] Culver B Itkin A Pischel K Case report and review of minocycline-induced cutaneous polyarteritis nodosa. Arthritis Rheum 2005;53:468–70. 15934105 10.1002/art.21186

[17] Tan CD Smith A Rodriguez ER Systemic necrotizing vasculitis induced by isoniazid. Cardiovasc Pathol 2014;23:181–2. 24508138 10.1016/j.carpath.2014.01.002

[18] Kim JH Moon JI Kim JE Cutaneous leukocytoclastic vasculitis due to anti-tuberculosis medications, rifampin and pyrazinamide. Allergy Asthma Immunol Res 2010;2:55–8. 20224679 10.4168/aair.2010.2.1.55 PMC2831607

[19] Cooper AM Cell-mediated immune responses in tuberculosis. Annu Rev Immunol 2009;27:393–422. 19302046 10.1146/annurev.immunol.021908.132703 PMC4298253

[20] Lyadova IV Panteleev AV Th1 and Th17 cells in tuberculosis: protection, pathology, and biomarkers. Mediators Inflamm 2015;2015:854507. 26640327 10.1155/2015/854507 PMC4657112

